# Therapeutic outcomes and prognostic determinants of neoadjuvant chemotherapy plus interval debulking surgery compared with primary debulking surgery in advanced-stage epithelial ovarian cancer

**DOI:** 10.3389/fonc.2026.1893041

**Published:** 2026-09-30

**Authors:** Yan Ma, Mengli Xu, Qi Zhang, Kamalibek Markya, Li Li

**Affiliations:** Department of Gynecology, Affiliated Tumor Hospital of the Third Clinical Medical College, Xinjiang Medical University, Urumqi, Xinjiang Uygur, China

**Keywords:** advanced epithelial ovarian cancer, comparison, NACT-IDS, PDS, prognostic factors, treatment efficacy

## Abstract

**Objective:**

To compare the therapeutic outcomes of neoadjuvant chemotherapy followed by interval debulking surgery (NACT-IDS) and primary debulking surgery (PDS) in patients with advanced epithelial ovarian cancer (AEOC) and to evaluate factors associated with prognosis.

**Methods:**

This retrospective study included 97 patients with AEOC treated between April 2015 and July 2020. According to treatment strategy, 36 patients underwent PDS and 61 underwent NACT-IDS. Overall survival (OS), progression-free survival (PFS), and perioperative outcomes were compared between the two groups. Kaplan–Meier analysis and Cox proportional hazards regression were used to evaluate survival outcomes. Additional adjusted models were used to account for baseline imbalance. Because CA-125 normalization occurred after treatment initiation, it was defined as a serum CA-125 concentration of ≤35 U/mL and analyzed as a time-dependent covariate.

**Results:**

Significant baseline differences were observed between the groups in age, BMI, baseline CA-125 level, and FIGO stage (all P<0.05). During a median follow-up of 43 months [95% CI (39.87, 50.32)], 53 of 97 patients (54.64%) died. Median OS was 41 months in the PDS group and 43 months in the NACT-IDS group, and median PFS was 20 months in both groups. No significant differences were found in unadjusted OS or PFS between the groups (OS: log-rank P = 0.484; PFS: log-rank P = 0.410). After adjustment for age, BMI, log-transformed baseline CA-125 level, and FIGO stage, treatment strategy was not associated with OS [adjusted HR = 0.88, 95% CI (0.49, 1.58), P = 0.667] or PFS [adjusted HR = 1.07, 95% CI (0.64, 1.79), P = 0.798]. Patients who achieved complete cytoreduction (R0) had longer median OS than those with incomplete cytoreduction (R1+R2) [43 vs. 38 months, P = 0.025] and longer median PFS [22 vs. 18 months, P = 0.040]. After adjustment, NACT-IDS remained associated with shorter operation time, lower intraoperative blood loss, earlier initiation of postoperative chemotherapy, and a lower likelihood of drainage-tube placement, whereas its association with postoperative complications was no longer statistically significant. Residual disease was associated with higher mortality [adjusted HR = 2.61, 95% CI 1.10–6.18, P = 0.029]. CA-125 normalization was associated with lower hazards of death [time-dependent adjusted HR = 0.59, 95% CI 0.35–0.99, P = 0.047] and progression or death [time-dependent adjusted HR = 0.63, 95% CI 0.42–0.95, P = 0.026].

**Conclusion:**

In this exploratory single-center retrospective non-randomized cohort, NACT-IDS was associated with shorter operation time, lower intraoperative blood loss, earlier initiation of postoperative chemotherapy, and less frequent drainage-tube placement than PDS, but not with a significantly lower adjusted postoperative complication rate or with OS or PFS. These findings should not be interpreted as evidence of equivalent survival efficacy or causal treatment benefit. Residual disease was associated with poorer survival, whereas documented CA-125 normalization showed a favorable time-dependent prognostic association.

## 1 Introduction

Advanced epithelial ovarian cancer (AEOC) is one of the most aggressive gynecologic malignancies, ranking highest in incidence and mortality among female reproductive system tumors worldwide (1). Due to the lack of early symptoms and effective screening methods, most patients are diagnosed at an advanced stage, resulting in poor prognosis (2). The standard treatment regimen includes primary debulking surgery (PDS) combined with postoperative adjuvant chemotherapy (3).

However, with the advancement of tumor biology research and optimization of treatment strategies in recent years, neoadjuvant chemotherapy (NACT) combined with interval debulking surgery (IDS) has gradually become a feasible alternative, especially for patients not suitable for immediate surgery (4).

The goal of PDS is to achieve complete gross cytoreduction, defined as no visible residual disease (R0). However, due to extensive tumor spread, the surgery is challenging, and some patients experience high postoperative complications or cannot tolerate surgery, limiting treatment efficacy (5). NACT-IDS, as an emerging treatment strategy, aims to shrink the tumor through preoperative chemotherapy, followed by IDS to improve the likelihood of complete cytoreduction and reduce surgical morbidity. This strategy is particularly suitable for patients with high tumor burden who cannot tolerate initial surgery (6). Although current clinical studies (7, 8) have reported the application efficacy of NACT-IDS and PDS in treating AEOC, there is still controversy regarding the comparison of their efficacy and prognostic factors. Therefore, this study aims to retrospectively analyze the clinical data of advanced epithelial ovarian cancer patients treated in our hospital, compare the efficacy of NACT-IDS and PDS, and explore the key factors influencing prognosis, providing a basis for clinical treatment decision-making.

## 2 Materials and methods

This retrospective study involving human participants was approved by the Ethics Committee of the Affiliated Cancer Hospital of Xinjiang Medical University (Approval No. FKZLK-LC1503; approval date: 2015-09.01). The study was conducted in accordance with the principles of the Declaration of Helsinki and its later amendments. Because this study retrospectively analyzed existing clinical data and did not involve additional intervention, the requirement for written informed consent was waived by the Ethics Committee of the Affiliated Cancer Hospital of Xinjiang Medical University. All patient data were anonymized before analysis.

### 2.1 Basic information

A retrospective analysis was conducted on the clinical data of 97 AEOC patients treated in our hospital from April 2015 to July 2020. This included general pathological indicators, preoperative and postoperative clinical data, and follow-up results. Inclusion criteria: ① patients aged ≥18 years and <80 years; ② patients with pathologically confirmed primary epithelial ovarian cancer (9); ③ patients with FIGO stage III–IV (10); ④ patients treated in our hospital with either NACT-IDS plus postoperative chemotherapy or PDS plus postoperative chemotherapy; ⑤ patients with complete data available for analysis. Primary fallopian tube carcinoma and primary peritoneal carcinoma were not included in this cohort. Exclusion criteria: ① metastatic tumors to the ovary from non-ovarian primary malignancies or ovarian cancer combined with synchronous primary malignancies of other organs; ② severe organ disease or immunodeficiency diseases; ③ severe hematological diseases; ④ history of previous chemotherapy; ⑤ severe infection or bleeding tendencies; ⑥ allergic reactions or contraindications to the drugs or methods used in this study; ⑦ cognitive disorders, consciousness disorders, and/or mental illnesses. Patients with FIGO stage IV AEOC were not excluded if the primary origin was confirmed as epithelial ovarian cancer.

Information on BRCA1/2 mutation status, homologous recombination deficiency (HRD) status, maintenance therapy, bevacizumab use, and PARP inhibitor use was reviewed when available. However, because these variables were not routinely tested or systematically recorded in all patients during the study period from 2015 to 2020, the data were incomplete and were not included as covariates in the main survival models.

### 2.2 Grouping

In this study, all 97 patients with AEOC underwent a comprehensive evaluation of general condition, surgical tolerance, tumor burden, and disease extent before treatment. Treatment allocation was not randomized but was determined according to clinical assessment and physician-patient decision-making. After initial assessment, 36 patients were considered suitable for primary debulking surgery (PDS), followed by postoperative adjuvant chemotherapy after adequate recovery. The remaining 61 patients were considered unsuitable for immediate PDS and received neoadjuvant chemotherapy (NACT) before interval debulking surgery (IDS). The standard first-line chemotherapy regimen was platinum-taxane chemotherapy. In most patients, paclitaxel 175 mg/m² plus carboplatin at an area under the curve of 5 was administered every 21 days, with dose adjustment according to hematologic toxicity, renal function, and general physical condition. Patients in the NACT-IDS group generally received 2–4 cycles of NACT before IDS. The number of NACT cycles was determined by clinical response, patient condition, and operability assessment rather than by a predefined comparative protocol. After each chemotherapy cycle, treatment response and operability were reassessed based on gynecologic examination, serum CA-125 level, general condition, organ function, and contrast-enhanced pelvic and abdominal CT or MRI. IDS was considered when imaging and clinical assessment suggested tumor shrinkage or stable disease, the patient had acceptable surgical tolerance, and complete or near-complete cytoreduction was considered achievable. Diagnostic laparoscopy was not routinely performed in all patients but was considered selectively when imaging findings were insufficient for surgical decision-making. Both PDS and IDS were performed with the goal of complete gross cytoreduction whenever feasible. Surgical procedures included total hysterectomy, bilateral salpingo-oophorectomy, omentectomy, peritoneal lesion resection, lymph node assessment or dissection, and bowel, diaphragmatic, splenic, or upper abdominal procedures when required. All operations were performed by senior gynecologic oncology surgeons at the Affiliated Cancer Hospital of Xinjiang Medical University. Multidisciplinary assistance from gastrointestinal, hepatobiliary, or thoracic surgeons was requested when extensive upper abdominal or bowel procedures were needed. Because of the retrospective design, formal surgical-complexity scores and sufficiently complete procedure-level data for quantitative adjustment were not consistently available. Postoperatively, patients were followed up regularly through inpatient records, outpatient visits, and telephone follow-up.

### 2.3 Evaluation indicators

The primary evaluation indicator in this study was overall survival (OS), with secondary indicators including progression-free survival (PFS), postoperative complications, and residual disease. OS was defined as the time from the start of treatment to death from any cause. PFS was defined as the time from the start of PDS or NACT to the first tumor recurrence, tumor progression, or death. Serum CA-125 was measured at treatment initiation and repeatedly during chemotherapy. CA-125 normalization was defined as a serum concentration of ≤35 U/mL according to the upper limit of normal used by the hospital laboratory. The date of the first documented normal CA-125 value was recorded for each patient. Because CA-125 normalization occurred after treatment initiation, it was not treated as a fixed baseline characteristic. For OS and PFS survival data, censoring was defined as patients still alive at the last outpatient or telephone follow-up, with the follow-up cut-off date being January 2024.

Postoperative complications were redefined as surgery-related adverse events occurring within 30 days after surgery or during the index hospitalization, whichever was longer. Complications were classified according to the Clavien-Dindo classification system. Grade I–II complications were defined as minor complications requiring conservative or pharmacological treatment, whereas grade III or higher complications were considered major complications requiring surgical, endoscopic, radiological intervention, intensive care management, or resulting in death. In addition, intraoperative or postoperative blood transfusion, 30-day readmission, reoperation, unplanned intensive care unit (ICU) admission, postoperative length of stay, and 30- and 90-day mortality were collected from medical records.

Residual disease status was assessed according to the amount of visible tumor remaining after cytoreductive surgery. In line with current ovarian cancer literature, complete cytoreduction was defined as R0, namely 0 mm/no visible residual disease, and was considered the most clinically relevant cytoreductive endpoint. R1 was defined as visible residual disease <1 cm, and R2 was defined as visible residual disease ≥1 cm. Because any visible residual disease may adversely affect prognosis, R1 and R2 were combined as incomplete cytoreduction (R1+R2) in the main survival analyses. Detailed surgical records were reviewed, and residual disease status was classified according to these criteria.

### 2.4 Statistical analysis

Statistical analysis was performed using SPSS 24.0. The normality of continuous variables was assessed using the Shapiro-Wilk test and visual inspection of histograms. Normally distributed continuous variables were expressed as mean ± standard deviation and compared using independent-samples t tests. Skewed continuous variables were expressed as median (interquartile range, IQR) and compared using the Mann-Whitney U test. Categorical variables were compared using the chi-square test, corrected chi-square test, or Fisher’s exact test, as appropriate. Because baseline CA-125 was right-skewed, it was reported as median (IQR), compared using the Mann-Whitney U test, and log-transformed before regression analyses. Survival analysis was conducted using Kaplan-Meier survival curves, and exact log-rank P values were reported for between-group comparisons.

Prognostic factors were analyzed using Cox proportional hazards models. To limit overfitting in this relatively small cohort, covariates in the multivariable prognostic models were prespecified according to clinical relevance rather than selected solely on the basis of univariable P values. Age was entered as a continuous covariate (per 1-year increase), whereas FIGO stage, treatment strategy, and residual disease status were included as categorical covariates in the parsimonious prognostic models. FIGO stage was retained because of its established clinical prognostic relevance, irrespective of its univariable P value. X-Tile 3.6.1 software was used only to identify exploratory cut-off values for the presentation of univariable analyses. Accordingly, the X-Tile-derived age cut-off of 69 years was retained only for the exploratory univariable analysis and was not used in the primary multivariable models.

To reduce guarantee-time and immortal-time bias, CA-125 normalization was analyzed as a time-dependent covariate in an extended Cox proportional hazards model. CA-125 status was coded as 0 from treatment initiation until the date of the first documented value ≤35 U/mL and as 1 thereafter. Patients who died, experienced disease progression, or discontinued chemotherapy before documented normalization contributed follow-up time only to the non-normalized state. Missing CA-125 values were not imputed, and patients without any post-baseline CA-125 measurement were excluded only from the CA-125-specific analysis. The time-dependent model was adjusted for treatment strategy, residual disease status, age as a continuous covariate, and FIGO stage. The proportional hazards assumption was assessed using time-dependent covariate tests by adding covariate × ln(time) interaction terms; P>0.05 was considered to indicate no violation of the proportional hazards assumption.

To reduce the influence of baseline imbalance and confounding by indication, additional adjusted analyses were performed to evaluate the association between treatment strategy and clinical outcomes. Treatment strategy (NACT-IDS vs. PDS) was entered as the main exposure, and age, BMI, log-transformed baseline CA-125 level, and FIGO stage were included as covariates. Cox regression was used for OS and PFS, multivariable linear regression was used for continuous perioperative outcomes, and multivariable logistic regression was used for binary perioperative outcomes. Because of the relatively small sample size and the marked imbalance in FIGO stage distribution between groups, propensity-score matching or inverse probability weighting was not performed to avoid excessive loss of cases and unstable estimates. The time-dependent CA-125 prognostic model and the treatment-strategy outcome models were specified separately because they addressed different research questions and therefore used different clinically prespecified covariate sets. All tests were two-sided, and P<0.05 was considered statistically significant.

## 3 Results

### 3.1 Comparison of baseline data

Significant baseline differences were observed between the PDS and NACT-IDS groups in age, BMI, baseline CA-125 level, and FIGO stage (all P<0.05), with the NACT-IDS group including older patients, higher CA-125 levels, lower BMI, and a higher proportion of FIGO stage IV disease. These findings indicated baseline imbalance and potential treatment-selection bias between the two groups, see **Table 1**.

**Table 1:** Comparison of baseline data.

Variable	PDS group (n=36)	NACT-IDS group (n=61)	*t/x²/Z*	*P*
Initial treatment age (years)	54.89 ± 10.76	59.34 ± 9.37	2.137	0.035
BMI (kg/m²)	23.47 ± 3.58	21.06 ± 3.72	3.125	0.002
CA- 125 (IU/mL)	780.50 (586.25–1050.75)	1578.00 (1156.50–2106.25)	-6.184	<0.001
Hypertension	–	–	0.002	0.964
Yes	9 (25.00)	15 (24.59)	–	–
No	27 (75.00)	46 (75.41)	–	–
Diabetes	–	–	0.848	0.357
Yes	10 (27.78)	12 (19.67)	–	–
No	26 (72.22)	49 (80.33)	–	–
HBSAg positive	–	–	0.013	0.906
Yes	5 (13.89)	9 (14.75)	–	–
No	31 (86. 11)	52 (85.25)	–	–
Previous surgery history	–	–	0.175	0.675
Yes	9 (25.00)	13 (21.31)	–	–
No	27 (75.00)	48 (78.69)	–	–
Affected ovary	–	–	0.041	0.839
Unilateral	14 (38.89)	25 (40.98)	–	–
Bilateral	22 (61. 11)	36 (59.02)	–	–
Ascites	–	–	0.203	0.651
Yes	20 (55.56)	31 (50.82)	–	–
No	16 (44.44)	30 (49. 18)	–	–
Pleural effusion	–	–	0.114	0.735
Yes	1 (2.78)	4 (6.56)	–	–
No	35 (97.22)	57 (93.44)	–	–
Histological type	–	–	1.259	0.261
Serous carcinoma	35 (97.22)	54 (88.52)	–	–
Non-serous carcinoma	1 (2.78)	7 (11.48)	–	–
Pathological grade	–	–	2.056	0.151
Low to moderate grade	2 (5.56)	11 (18.03)	–	–
High grade	34 (94.44)	50 (81.97)	–	–
FIGO stage	–	–	12.171	<0.001
Stage III	30 (83.33)	29 (47.54)	–	–
Stage IV	6 (16.67)	32 (52.46)	–	–

Normally distributed continuous variables are presented as mean ± SD; skewed continuous variables are presented as median (IQR); categorical variables are presented as n (%). CA-125 was compared using the Mann-Whitney U test. Statistical tests are indicated in the Statistic/test column. The independent-samples t test was used for normally distributed continuous variables, the Mann-Whitney U test was used for skewed continuous variables, the Pearson chi-square test or continuity-corrected chi-square test was used for categorical variables as appropriate. All P values were two-sided. P<0.001 was reported when the calculated P value was less than 0.001.

### 3.2 Comparison of OS and PFS

By the follow-up deadline, 53 out of 97 patients (54.64%) had died, with a median follow-up time of 43 months [95% CI (39.87, 50.32)]. The median OS for the PDS group and the NACT-IDS group were 41 months [95% CI (27.24, 55.36)] and 43 months [95% CI (39.41, 47.89)] respectively, and the median PFS for both groups was 20 months. In the unadjusted Kaplan-Meier analysis, there were no statistically significant differences in median OS or median PFS between the PDS and NACT-IDS groups (OS: log-rank P = 0.484; PFS: log-rank P = 0.410), see **Figures 1**, **2**.

**Figure 1:**
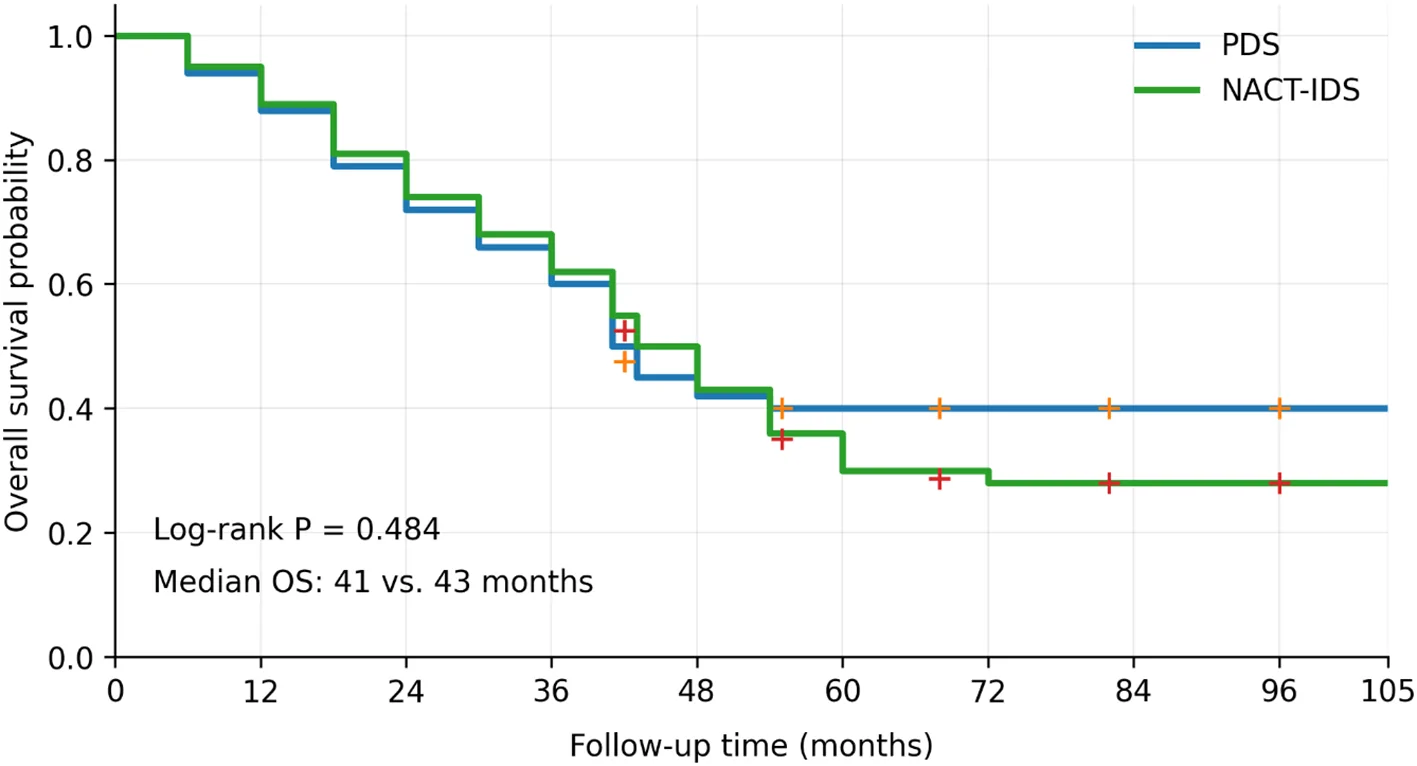
Kaplan-Meier curve of overall survival according to treatment strategy. PDS, primary debulking surgery; NACT-IDS, neoadjuvant chemotherapy followed by interval debulking surgery. The x-axis was restricted to 0–105 months according to the actual enrollment period and follow-up cut-off date. The P value was calculated using the log-rank test. Numbers at risk at 0, 24, 48, 72, and 96 months were as follows: PDS, 36, 26, 15, 12, and 8; NACT-IDS, 61, 45, 23, 15, and 10.

**Figure 2:**
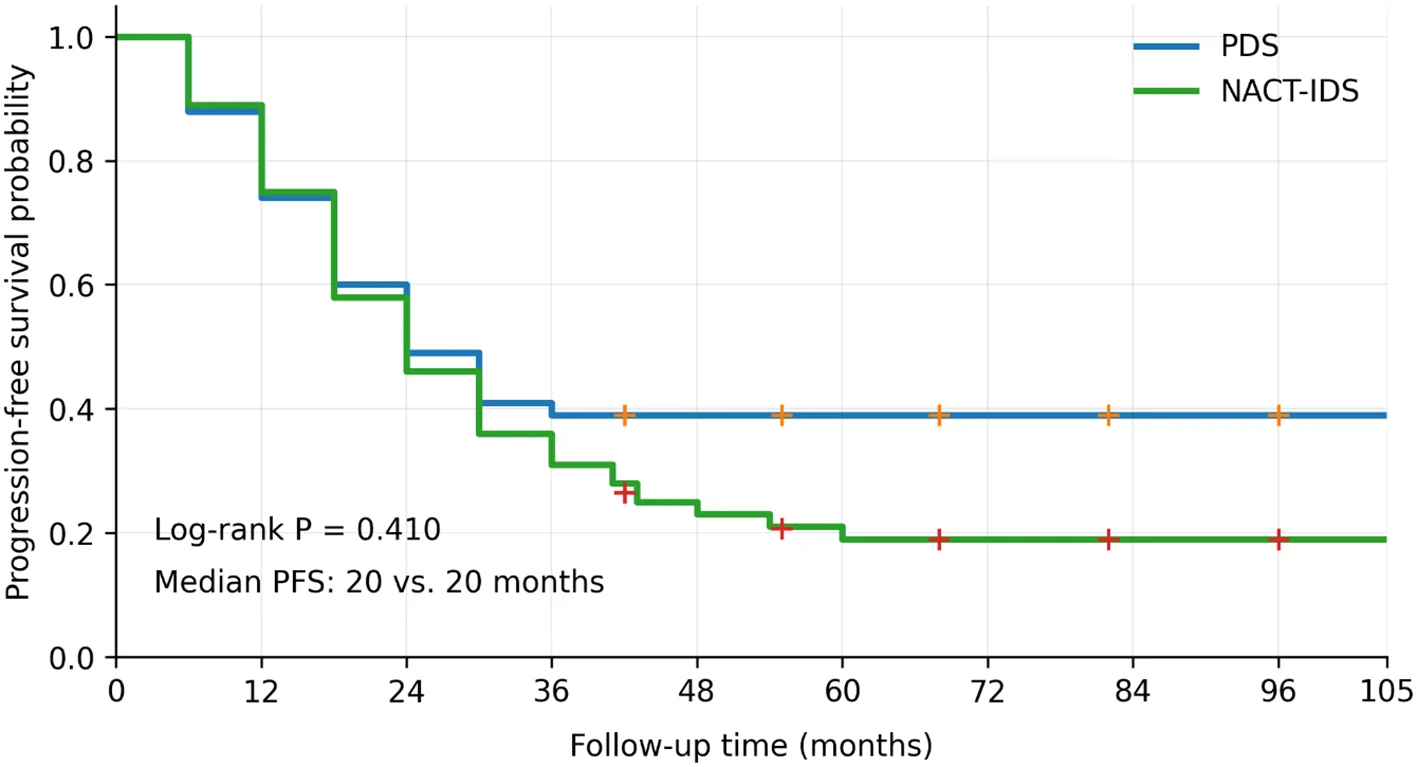
Kaplan-Meier curve of progression-free survival according to treatment strategy. PDS, primary debulking surgery; NACT-IDS, neoadjuvant chemotherapy followed by interval debulking surgery. The x-axis was restricted to 0–105 months according to the actual enrollment period and follow-up cut-off date. The P value was calculated using the log-rank test. Numbers at risk at 0, 24, 48, 72, and 96 months were as follows: PDS, 36, 18, 10, 7, and 5; NACT-IDS, 61, 29, 11, 9, and 5.

Patients who achieved complete cytoreduction (R0, 0 mm/no visible residual disease) had longer median OS than those with incomplete cytoreduction (R1+R2, any visible residual disease) (43 months vs. 38 months), and median PFS was also longer in the R0 group than in the R1+R2 group (22 months vs. 18 months).

The differences between the groups were statistically significant in the log-rank test (OS: P = 0.025; PFS: P = 0.040), see **Figures 3**, **4**. All Kaplan-Meier curves were rechecked and replotted with the x-axis restricted to 0–105 months according to the actual enrollment period and follow-up cut-off date.

**Figure 3:**
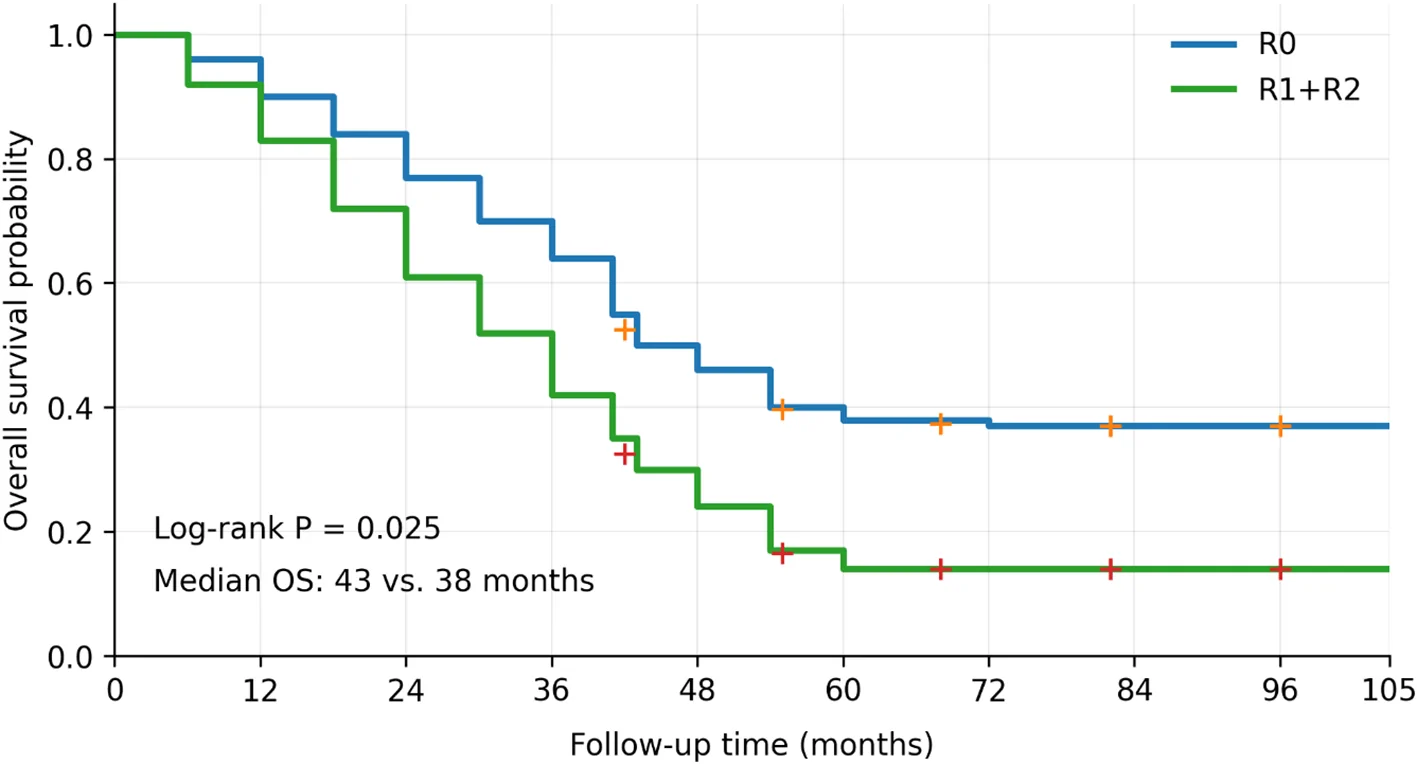
Kaplan-Meier curve of overall survival according to residual disease status. R0 indicates no visible residual tumor after cytoreductive surgery; R1+R2 indicates visible residual disease after cytoreductive surgery. The x-axis was restricted to 0–105 months according to the actual enrollment period and follow-up cut-off date. The P value was calculated using the log-rank test. Numbers at risk at 0, 24, 48, 72, and 96 months were as follows: R0, 68, 53, 31, 23, and 15; R1+R2, 29, 18, 7, 4, and 3.

**Figure 4:**
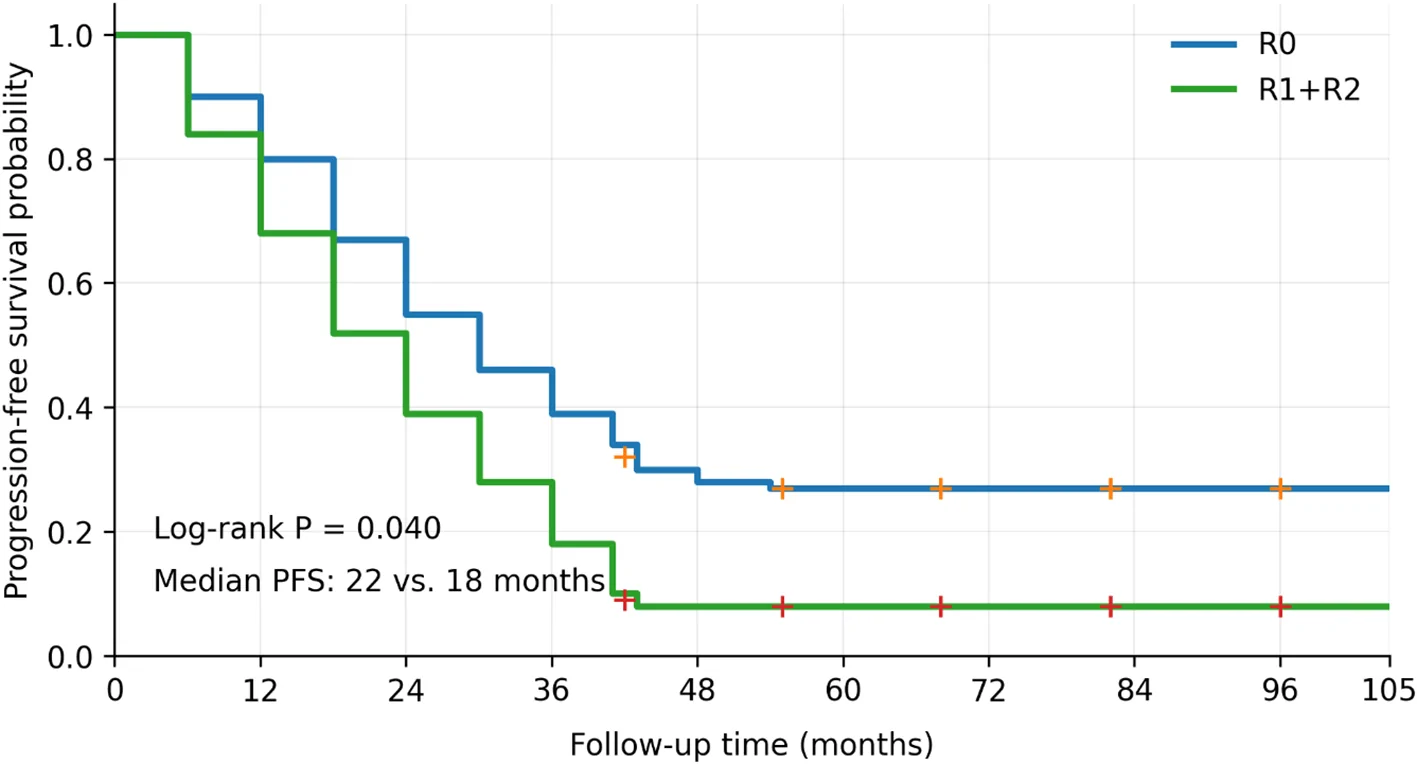
Kaplan-Meier curve of progression-free survival according to residual disease status. R0 indicates no visible residual tumor after cytoreductive surgery; R1+R2 indicates visible residual disease after cytoreductive surgery. The x-axis was restricted to 0–105 months according to the actual enrollment period and follow-up cut-off date. The P value was calculated using the log-rank test. Numbers at risk at 0, 24, 48, 72, and 96 months were as follows: R0, 68, 36, 19, 15, and 9; R1+R2, 29, 11, 2, 1, and 1.

### 3.3 Comparison of perioperative outcomes

In the unadjusted comparison, compared with the PDS group, the NACT-IDS group had lower intraoperative blood loss, shorter operation time, lower incidence of 30-day postoperative complications, lower proportion of patients with indwelling drainage tubes, and shorter time to initiation of postoperative chemotherapy (P<0.05). The recorded postoperative complications mainly included wound infection or poor wound healing, pelvic or abdominal infection/postoperative fever requiring antibiotics, postoperative ileus or gastrointestinal dysfunction, lymphatic leakage or prolonged drainage, pulmonary infection or pleural effusion requiring treatment, and intra-abdominal bleeding or abscess requiring intervention. Most complications were Clavien-Dindo grade I–II. No Clavien-Dindo grade IV–V complications, 30-day mortality, or 90-day mortality occurred in either group. There were no significant differences in residual disease rate, adverse chemotherapy reactions, 30-day readmission, reoperation within 30 days, or unplanned ICU admission between the two groups (P>0.05), see **Table 2**.

**Table 2:** Comparison of perioperative outcomes and standardized postoperative morbidity.

Outcome	PDS group (n=36)	NACT-IDS group (n=61)	*t/x²*	P
Operation time (min)	284.76 ± 81.59	205.97 ± 46.32	6.075	<0.001
Intraoperative blood loss (mL)	597.84 ± 143.71	239.85 ± 53.43	17.557	<0.001
Blood transfusion	17 (47.22)	11 (18.03)	9.260	0.002
Indwelling drainage tube	25 (69.44)	23 (37.70)	9.123	0.002
Postoperative length of stay (days)	13.72 ± 4.26	10.84 ± 3.18	3.497	0.001
Time to start chemotherapy postoperatively (days)	18.43 ± 4.39	9.36 ± 1.27	15.145	<0.001
Any 30-day postoperative complication	10 (27.78)	7 (11.48)	4.162	0.041
Clavien-Dindo grade I–II	7 (19.44)	6 (9.84)	—	—
Clavien-Dindo grade III	3 (8.33)	1 (1.64)	—	—
Clavien-Dindo grade IV–V	0 (0.00)	0 (0.00)	—	—
30-day readmission	3 (8.33)	2 (3.28)	—	0.359
Reoperation within 30 days	2 (5.56)	1 (1.64)	—	0.555
Unplanned ICU admission	3 (8.33)	1 (1.64)	—	0.136
30-day mortality	0 (0.00)	0 (0.00)	—	—
90-day mortality	0 (0.00)	0 (0.00)	—	—
Residual disease	14 (38.89)	15 (24.59)	2.208	0.137
Adverse chemotherapy reactions	29 (80.56)	52 (85.25)	0.361	0.547

Continuous variables are presented as mean ± SD, and categorical variables are presented as n (%). The independent-samples t test was used for normally distributed continuous variables. The Pearson chi-square test was used for categorical variables, and Fisher’s exact test was used when expected cell counts were small. Postoperative complications were defined as surgery-related adverse events occurring within 30 days after surgery or during the index hospitalization, whichever was longer, and were graded according to the Clavien-Dindo classification. P values were two-sided. P<0.001 was reported when the calculated P value was less than 0.001. ICU, intensive care unit; PDS, primary debulking surgery; NACT-IDS, neoadjuvant chemotherapy followed by interval debulking surgery.

### 3.4 Cox regression analysis

To minimize overfitting, the primary prognostic analysis used a parsimonious multivariable Cox model with clinically prespecified covariates, including age modeled as a continuous variable, FIGO stage, treatment strategy, and residual disease status (**Table 3**). In this model, residual disease remained significantly associated with OS after adjustment for treatment strategy and the other prespecified covariates. Patients with R1/R2 disease had a higher risk of death than those who achieved R0 cytoreduction [adjusted HR = 2.61, 95% CI (1.10, 6.18), P = 0.029]. Residual disease was not significantly associated with PFS [adjusted HR = 1.31, 95% CI (0.65, 2.65), P = 0.451]. Treatment strategy was not independently associated with OS [adjusted HR = 0.90, 95% CI (0.49, 1.67), P = 0.736] or PFS [adjusted HR = 1.06, 95% CI (0.63, 1.80), P = 0.828] (**Table 4**).

**Table 3:** Exploratory univariable Cox regression analyses of OS and PFS in patients with advanced epithelial ovarian cancer.

Variable	OS		PFS	
	HR (95% CI)	P	HR (95% CI)	P
Age (>69 years vs. ≤69 years)	2.11 (1.03, 4.35)	0.046	2.05 (1.06, 3.97)	0.030
FIGO stage (Stage IV vs. Stage III)	1.62 (0.94, 2.69)	0.079	1.64 (1.03, 2.58)	0.032
Serous carcinoma (yes vs. no)	1.02 (0.37, 2.81)	0.986	0.84 (0.32, 2.35)	0.731
Ascites (yes vs. no)	1.12 (0.63, 1.85)	0.746	1.10 (0.71, 1.73)	0.701
Positive para-aortic lymph nodes (yes vs. no)	0.42 (0.12, 1.34)	0.143	0.58 (0.23, 1.46)	0.259
NACT cycles (>2 courses vs. ≤2 courses)	0.83 (0.41, 1.54)	0.509	0.77 (0.43, 1.38)	0.385
Treatment method (NACT-IDS vs. PDS)	0.94 (0.50, 1.63)	0.774	1.21 (0.75, 1.96)	0.489
Residual disease (R1+R2 vs. R0)	1.89 (1.06, 3.27)	0.027	1.67 (1.02, 2.69)	0.046

P values were calculated using the Wald test in the Cox proportional hazards regression models. The comparison direction for each HR is indicated in parentheses, with the second category used as the reference group. All P values were two-sided. P<0.001 was reported when the calculated P value was less than 0.001. HR, hazard ratio; CI, confidence interval; OS, overall survival; PFS, progression-free survival. These univariable analyses were exploratory and were not used as the sole criterion for covariate selection in the primary multivariable models.

**Table 4:** Multivariable Cox regression analysis of prognostic factors in patients with advanced epithelial ovarian cancer.

Variable	OS		PFS	
	HR (95% CI)	P	HR (95% CI)	P
Age (per 1-year increase)	1.01 (0.98, 1.04)	0.512	1.02 (0.99, 1.05)	0.187
FIGO stage (Stage IV vs. Stage III)	1.61 (0.72, 3.61)	0.247	1.29 (0.63, 2.64)	0.486
Treatment strategy (NACT-IDS vs. PDS)	0.90 (0.49, 1.67)	0.736	1.06 (0.63, 1.80)	0.828
Documented CA-125 normalization, time-dependent	0.59 (0.35, 0.99)	0.047	0.63 (0.42, 0.95)	0.026
Residual disease (R1+R2 vs. R0)	2.61 (1.10, 6.18)	0.029	1.31 (0.65, 2.65)	0.451

The parsimonious multivariable models included age as a continuous covariate (per 1-year increase), together with FIGO stage, treatment strategy, and residual disease status as clinically prespecified covariates. CA-125 normalization was modeled as a time-dependent covariate and was adjusted for the same prespecified covariates, with age entered continuously. CA-125 status was coded as non-normalized before the first documented value ≤35 U/mL and as normalized thereafter. Patients who experienced an outcome before normalization contributed follow-up time only to the non-normalized state. The prognostic models presented in this table were distinct from the treatment-strategy outcome models presented in **Table 5**. P values were calculated using the Wald test. All P values were two-sided. HR, hazard ratio; CI, confidence interval; OS, overall survival; PFS, progression-free survival; PDS, primary debulking surgery; NACT-IDS, neoadjuvant chemotherapy followed by interval debulking surgery.

Post-baseline CA-125 measurements were available for 92 of the 97 patients, including 34 patients in the PDS group and 58 in the NACT-IDS group. The remaining five patients, including two in the PDS group and three in the NACT-IDS group, had no post-baseline CA-125 measurements and were therefore excluded from the CA-125-specific analysis. During follow-up, documented CA-125 normalization was observed in 64 patients, whereas 28 patients did not achieve normalization. Within the first four postoperative chemotherapy cycles, three patients died, six experienced disease progression, and four discontinued chemotherapy. These patients remained in the risk set until the corresponding event or censoring and contributed follow-up time only to the non-normalized state.

In the extended Cox model adjusted for treatment strategy, residual disease status, age as a continuous covariate, and FIGO stage, documented CA-125 normalization was associated with lower hazards of death [adjusted HR = 0.59, 95% CI (0.35, 0.99), P = 0.047] and progression or death [adjusted HR = 0.63, 95% CI (0.42, 0.95), P = 0.026]. These results support a favorable time-dependent prognostic association rather than establishing CA-125 normalization as a fixed independent prognostic factor. These results support a favorable time-dependent prognostic association rather than establishing CA-125 normalization as a fixed independent prognostic factor.

To further account for baseline imbalance between the PDS and NACT-IDS groups, additional adjusted analyses were performed with treatment strategy as the main exposure and age, BMI, log-transformed baseline CA-125 level, and FIGO stage as covariates. After adjustment, treatment strategy was not independently associated with OS or PFS. NACT-IDS remained associated with shorter operation time, lower intraoperative blood loss, earlier initiation of postoperative chemotherapy, and a lower likelihood of indwelling drainage-tube placement. However, the association between NACT-IDS and postoperative complications was attenuated and was no longer statistically significant after adjustment. These results are presented in **Table 5** and indicate that the unadjusted survival comparisons should be interpreted cautiously in view of the baseline imbalance and potential treatment-selection bias. The proportional hazards assumption was assessed for all Cox models, and no significant violations were identified.

**Table 5:** Adjusted analysis of treatment strategy for survival and perioperative outcomes.

Outcome	Model	Effect estimate for NACT-IDS vs. PDS	95% CI	P value
OS	Adjusted Cox regression, HR	0.88	0.49–1.58	0.667
PFS	Adjusted Cox regression, HR	1.07	0.64–1.79	0.798
Operation time	Adjusted linear regression, β (min)	-64.30	-91.80 to -36.80	<0.001
Intraoperative blood loss	Adjusted linear regression, β (mL)	-316.50	-379.20 to -253.70	<0.001
Time to start postoperative chemotherapy	Adjusted linear regression, β (days)	-8.10	-9.80 to -6.40	<0.001
Postoperative complications	Adjusted logistic regression, OR	0.42	0.14–1.24	0.118
Indwelling drainage tube	Adjusted logistic regression, OR	0.35	0.14–0.87	0.024

The adjusted models included age, BMI, log-transformed baseline CA-125 level, and FIGO stage as covariates. P values were derived from Wald tests for Cox and logistic regression models and from t tests for linear regression models. All P values were two-sided. P<0.001 was reported when the calculated P value was less than 0.001. HR, hazard ratio; OR, odds ratio; CI, confidence interval; OS, overall survival; PFS, progression-free survival; PDS, primary debulking surgery; NACT-IDS, neoadjuvant chemotherapy followed by interval debulking surgery.

## 4 Discussion

Ovarian cancer is a highly heterogeneous malignancy with complex biological behavior. Improving the prognosis of patients with advanced ovarian cancer remains a major clinical challenge (11). Among the many influencing factors, postoperative residual lesions are considered crucial in determining the survival prognosis of patients with advanced ovarian cancer. Previous studies (12) have shown that patients who achieve R0, defined as no visible residual disease after cytoreductive surgery, have better OS than those who do not achieve complete cytoreduction. Therefore, in the present study, R0 was emphasized as the key cytoreductive endpoint, whereas R1 and R2 were retained only to describe the extent of visible residual disease and were combined as incomplete cytoreduction in the main survival analyses. In the present study, patients who achieved R0 had median OS and PFS extended by 5 and 4 months, respectively, compared with those with incomplete cytoreduction (R1+R2). The association between residual disease and OS remained significant after adjustment for age, FIGO stage, and treatment strategy. This finding is consistent with previous research (13, 14) and supports the prognostic importance of complete cytoreduction in newly diagnosed AEOC. However, some studies (15) have reported that achieving R0 may require more extensive and complex surgery, which can increase the risk of postoperative complications. Nevertheless, other studies (16, 17) suggest that the survival benefit of extensive cytoreductive surgery may partly offset the risk of perioperative morbidity. In the PDS setting, complete gross cytoreduction may require ultra-radical procedures involving the upper abdomen, liver area, diaphragm, bowel, spleen, or thoracic region, and therefore often depends on multidisciplinary surgical collaboration. Rosati et al. recently reported the feasibility and operative outcomes of surgery in the liver area in advanced ovarian cancer, highlighting the importance of specialized multidisciplinary surgical management for achieving cytoreductive goals (18). Therefore, when PDS is considered, patients should ideally be treated in experienced gynecologic oncology centers with access to multidisciplinary surgical support, so as to maximize the likelihood of complete cytoreduction while controlling perioperative morbidity. Changes in CA-125 levels during treatment have long been considered useful for prognostic evaluation (19). Because CA-125 normalization is a post-baseline event, treating it as a fixed covariate may introduce guarantee-time or immortal-time bias. We therefore analyzed documented CA-125 normalization as a time-dependent covariate. After adjustment for treatment strategy, residual disease status, age, and FIGO stage, CA-125 normalization remained associated with lower hazards of death and progression or death. However, this finding should be interpreted as a time-dependent prognostic association rather than evidence of an independent causal effect.

PDS and NACT-IDS are two major treatment strategies for advanced ovarian cancer, although the optimal selection of patients for NACT-IDS remains controversial. In 2012, the National Comprehensive Cancer Network (NCCN) first recommended in its ovarian cancer guidelines (20) that NACT-IDS could be considered for patients unsuitable for immediate surgery. A previous meta-analysis involving 1,220 patients with advanced ovarian cancer showed no significant difference in median OS between NACT-IDS and PDS (21). In the subgroup analysis of stage IV patients, the median OS of NACT-IDS was found to be about 3 months longer than that of PDS, with statistical significance. In the present study, patients in the NACT-IDS group were older, had higher CA-125 levels, and had a higher proportion of FIGO stage IV disease than those in the PDS group, suggesting that treatment allocation was influenced by baseline disease burden and surgical suitability. Therefore, the survival comparison between NACT-IDS and PDS should be interpreted cautiously. Although no significant differences in OS or PFS were observed in the unadjusted analysis and treatment strategy was not independently associated with OS/PFS after multivariable adjustment, these findings should be considered exploratory rather than evidence of equivalent survival efficacy, non-inferiority, or causal treatment benefit. In addition, contemporary prognostic and therapeutic factors, including BRCA1/2 mutation status, HRD status, maintenance therapy, bevacizumab use, and PARP inhibitor use, may influence PFS and OS. Because these data were incomplete in this retrospective cohort, they could not be incorporated into the adjusted models and should be considered potential unmeasured confounders. NACT-IDS may be a feasible option for selected patients who are unsuitable for immediate primary surgery, but larger prospective studies or well-adjusted multicenter cohorts are needed to further clarify the optimal selection of PDS versus NACT-IDS. The optimal number of NACT cycles and the timing of IDS remain clinically important but controversial issues. In the present study, patients in the NACT-IDS group generally received 2–4 cycles of NACT before IDS; however, this study was not designed to compare different NACT cycle numbers or to determine the optimal timing of IDS. Marchetti et al. reported in a propensity score-matched analysis that the number of NACT cycles may influence oncologic outcomes and should be considered when planning treatment strategies (22). Therefore, the decision to proceed to IDS should be individualized rather than interpreted as simply “as early as possible.” Another contemporary issue in the NACT-IDS setting is the potential role of minimally invasive surgery (MIS) for selected patients. MIS-IDS may reduce surgical trauma, perioperative morbidity, and recovery time, and may improve postoperative quality of life when complete cytoreduction can be safely achieved. However, MIS is not suitable for all patients with advanced ovarian cancer, and its use should be limited to carefully selected patients with favorable response to NACT, limited residual tumor burden, no radiologic evidence of disease requiring extensive upper abdominal or bowel resection, and a high likelihood of complete cytoreduction. Conte et al. developed a nomogram to predict the feasibility of MIS-IDS after NACT, suggesting that patient selection should integrate tumor distribution, CA-125 response, and surgeon or center experience (23). The ongoing LANCE trial is expected to provide prospective evidence regarding the oncologic safety and perioperative impact of MIS compared with laparotomy in selected patients undergoing IDS after NACT (24). More recently, Conte et al. emphasized that radiologic selection for MIS-IDS remains complex and may require combined assessment using imaging, diagnostic laparoscopy, or advanced tools (25). Therefore, MIS-IDS should be regarded as an emerging strategy for highly selected IDS candidates rather than as a general alternative to open cytoreductive surgery.

In terms of perioperative outcomes, the evaluated parameters included operation time, intraoperative blood loss, blood transfusion, drainage-tube placement, postoperative length of stay, standardized 30-day postoperative complications, readmission, reoperation, unplanned ICU admission, and 30- and 90-day mortality. No 30-day or 90-day perioperative deaths occurred in either group. In the unadjusted analysis, the NACT-IDS group had shorter operation time, lower intraoperative blood loss, a lower rate of blood transfusion, fewer postoperative complications, less frequent drainage-tube placement, and earlier initiation of postoperative chemotherapy than the PDS group, which was generally consistent with previous findings (26). After multivariable adjustment, NACT-IDS remained associated with shorter operation time, lower intraoperative blood loss, earlier initiation of postoperative chemotherapy, and less frequent drainage-tube placement. However, the association with postoperative complications was attenuated and was no longer statistically significant. Because formal surgical-complexity scores and sufficiently detailed procedure-level data were unavailable, the observed differences in operation time, blood loss, transfusion, and postoperative recovery may partly reflect differences in the extent and complexity of surgery, as well as clinical treatment selection, rather than the treatment strategy itself. The lower frequency of drainage-tube placement in the NACT-IDS group should therefore not be interpreted as direct evidence of less surgical trauma or faster recovery. Patients in the NACT-IDS group initiated postoperative chemotherapy approximately 9 days after surgery, compared with approximately 18 days in the PDS group. Although the timing of postoperative chemotherapy may be clinically relevant (27), the present study did not evaluate whether earlier initiation reduced recurrence or improved oncologic outcomes. Therefore, the observed perioperative associations should be interpreted cautiously in the context of baseline imbalance, treatment-selection bias, and unmeasured differences in surgical complexity. NACT-IDS may be considered a feasible treatment strategy for selected patients who are unsuitable for immediate PDS, but these findings should not be interpreted as evidence of a causal perioperative benefit.

In summary, residual disease was associated with poorer survival, whereas documented CA-125 normalization showed a favorable time-dependent prognostic association. In this exploratory single-center retrospective non-randomized cohort, NACT-IDS was associated with differences in selected perioperative parameters, including shorter operation time, lower intraoperative blood loss, earlier initiation of postoperative chemotherapy, and less frequent drainage-tube placement, while its adjusted association with postoperative complications was not statistically significant. However, these findings do not establish comparable survival efficacy or superiority of NACT-IDS, particularly in FIGO stage IV disease, because causal conclusions cannot be drawn from this retrospective study.

These findings should be interpreted in light of several limitations. First, the sample size was relatively small, which may affect the reliability and generalizability of the results and may limit the statistical power for some analyses. Second, this was a retrospective non-randomized study. Although an adjusted treatment-effect analysis was performed using multivariable models, patients in the NACT-IDS group were older, had higher CA-125 levels, lower BMI, and a higher proportion of FIGO stage IV disease. These differences suggest that patients with greater disease burden or lower suitability for immediate surgery were more likely to receive NACT-IDS. Therefore, treatment-selection bias and residual confounding could not be completely avoided. In addition, because of the limited sample size, more robust adjustment methods such as propensity-score matching or inverse probability weighting were not performed. The observed similarity in OS and PFS should therefore not be interpreted as evidence of equivalent efficacy between NACT-IDS and PDS. In addition, this study did not specifically evaluate the optimal number of NACT cycles or the optimal timing of IDS, and therefore no conclusion can be drawn regarding whether IDS should be performed earlier or later after NACT. The chronological and biological timing of postoperative CA-125 assessment also differed between the treatment groups because patients in the NACT-IDS group had received 2–4 cycles of chemotherapy before surgery. Although CA-125 normalization was analyzed as a time-dependent covariate and treatment strategy was included in the adjusted model, differences in cumulative chemotherapy exposure and the timing of CA-125 testing may still have introduced residual measurement-related bias. This study also did not separately analyze open IDS and MIS-IDS, and the clinical and imaging variables required for formal MIS candidacy assessment were incomplete; therefore, no conclusion can be drawn regarding the feasibility, safety, or oncologic outcomes of MIS-IDS in this cohort. Third, X-Tile-derived cut-offs were used only for exploratory univariable prognostic stratification and were not used in the primary multivariable models. Nevertheless, these data-driven thresholds may be susceptible to overfitting and require external validation. Fourth, complete molecular data, including BRCA1/2 mutation status and HRD status, were unavailable in some patients. Data on maintenance therapy, bevacizumab use, PARP inhibitor use, laparoscopy-based resectability scoring, and formal surgical-complexity scoring were also incomplete or unavailable. Detailed procedure-level data were also insufficient for quantitative adjustment; therefore, residual differences in surgical extent and complexity may have contributed to the observed differences in operative time, blood loss, transfusion, drainage-tube use, and postoperative recovery. These factors may influence PFS and OS and should be considered important unmeasured confounders. Fifth, this was a single-center study, which may limit the external validity of the findings. Future studies with larger sample sizes, multicenter designs, prospective data collection, and more complete molecular and treatment-related information are needed to further validate these findings and improve patient selection for PDS or NACT-IDS.

## Data Availability

The original contributions presented in the study are included in the article/supplementary material. Further inquiries can be directed to the corresponding author.
